# Authorship, titles and open access as drivers of citation performance in orthopaedics: a scientometric analysis

**DOI:** 10.1186/s10195-026-00911-z

**Published:** 2026-03-20

**Authors:** Filippo Migliorini, Raju Vaishya, Fabrizio Rivera, Jörg Eschweiler, Philipp Kobbe, Marcel Betsch, Francesco Oliva, Nicola Maffulli

**Affiliations:** 1 Department of Trauma and Reconstructive Surgery, University Hospital of Halle, Martin-Luther University Halle-Wittenberg, Ernst-Grube-Street 40, 06097 Halle (Saale), Germany; 2 Department of Orthopaedic and Trauma Surgery, Eifelklink St.Brigidia, 52152 Simmerath, Germany; 3 Department of Life Sciences, Health, and Health Professions, Link Campus University, 00165 Rome, Italy; 4 Department of Orthopaedics and Joint Replacement Surgery, Indraprastha Apollo Hospital, Sarita Vihar, 110076 New Delhi, India; 5 Department of Orthopaedics and Traumatology, Ospedale SS Annunziata, ASL CN1, Via Ospedali, 9, 12038 Savigliano, Italy; 6 Department of Orthopaedic and Trauma Surgery, University Hospital Erlangen, Erlangen, Germany; 7 Department of Sports Traumatology, Università Telematica San Raffaele, Rome, Italy; 8 Department of Trauma and Orthopaedic Surgery, Faculty of Medicine and Psychology, University La Sapienza, 00185 Rome, Italy; 9 School of Pharmacy and Bioengineering, Keele University Faculty of Medicine, Stoke On Trent, ST4 7QB UK; 10 Centre for Sports and Exercise Medicine, Barts and the London School of Medicine and Dentistry, Mile End Hospital, Queen Mary University of London, London, E1 4DG UK; 11 Department of Trauma and Reconstructive Surgery, BG Klinikum Bergmannstrost Halle GmbH, Halle(Saale), Germany

**Keywords:** Bibliometrics, Research impact, Collaboration, Scholarly publishing, Publication ethics, Academic visibility, Citation analysis

## Abstract

**Background:**

Bibliometric analyses are increasingly used to explore how scientific knowledge is created, disseminated, and perceived. In orthopaedics, research output has expanded rapidly over the past decade, yet the factors determining whether an article achieves wide visibility and scholarly impact remain poorly understood. Beyond the inherent quality of a study, elements such as authorship patterns, title construction, and open access (OA) availability may play an essential role in shaping citation performance. However, evidence in this field is still limited and sometimes contradictory, highlighting the need for large-scale, field-specific analyses.

**Methods:**

Orthopaedic publications from 2010 to 2020 were identified in Scopus using the keyword ‘orthopaedic’. After duplicate removal, 97,806 unique articles were included with complete data on authorship, titles, citation counts, study design, and OA status. Citation rates were normalised per year since publication. Associations between bibliographic features and citation performance were assessed using multiple linear regression, while differences across title styles and study designs were evaluated with comparative statistical testing. Exploratory modelling was performed to identify combinations of authorship and title characteristics linked to the highest predicted citation rates.

**Results:**

Larger author teams were associated with higher citation rates (*β* = 0.108 citations/year per additional author, 95% confidence interval [CI] 0.103–0.114, *p* < 0.001). OA articles achieved a mean increase of 0.175 citations/year compared with non-OA (*p* = 0.001). Title length in characters correlated positively with citation rate (*β* = 0.023 per character, *p* < 0.001), whereas title length in words showed a negative association (*β* = −0.183 per word, *p* < 0.001). The presence of a colon (+0.314 citations/year, *p* < 0.001) or dash (+0.187, *p* = 0.001) increased citation performance, while question marks (−0.476, *p* < 0.001) and all-capital titles (mean 0.71 citations/year) reduced it. Regarding study design, network meta-analyses achieved the highest citation rate (mean 6.64 citations/year), followed by systematic reviews (5.66), meta-analyses (5.08) and narrative reviews (4.81). Randomised controlled trials (3.90) and clinical trials (3.86) performed at an intermediate level, whereas observational studies (2.40), case series (1.79), technical notes (1.33), case reports (0.77), editorials (0.51) and commentaries (0.25) showed consistently lower citation performance (*p* < 0.0001).

**Conclusions:**

In orthopaedic research, collaboration, OA availability and concise, well-structured titles with selected punctuation contribute to higher citation performance, while unconventional title formatting reduces visibility. Although useful for optimising dissemination, ethical authorship practices and rigorous scientific standards remain more critical than citation metrics.

## Introduction

Bibliometric analysis has become increasingly important in understanding patterns of knowledge production, dissemination and impact across scientific disciplines [1–3]. In orthopaedics, where research output has expanded substantially over the past two decades, quantitative evaluation of publication characteristics offers valuable insights into the factors that may influence visibility and scholarly uptake [4–6]. Citation counts remain the most widely used proxy for academic impact, despite well-recognised limitations such as their susceptibility to field size, citation cultures and time since publication [7–9]. Consequently, there has been growing interest in identifying modifiable elements of research dissemination associated with higher citation rates, allowing investigators to optimise reach without compromising scientific integrity [10]. Among these elements, authorship patterns and title characteristics have emerged as recurrent topics of bibliometric inquiry [11, 12]. Multi-authored studies are often assumed to benefit from greater exposure through wider professional networks, potentially leading to higher citation rates [13, 14]. However, the relationship is not necessarily linear and may vary according to discipline and research design [15]. Title construction also influences discoverability in bibliographic databases, with features such as length, specificity and punctuation marks playing a role in attracting readership [12, 16]. However, the evidence in orthopaedics remains sparse and sometimes contradictory, particularly regarding the balance between brevity and informativeness, and whether specific stylistic devices confer measurable advantages. Open access (OA) publishing has recently introduced further complexity to the citation landscape [17, 18]. Several studies across biomedical fields have reported a modest citation advantage for OA articles, plausibly given the reduced access barriers and increased online sharing [18–21]. Hence, the magnitude of this effect and its persistence over time are debated. Moreover, the sustainability of OA models and the equity implications of high article processing charges continue to be points of contention in the academic community [22, 23]. Understanding these relationships in orthopaedics is not purely an academic exercise. Indeed, it has practical relevance for researchers aiming to maximise the dissemination and influence of their work within ethical and professional boundaries. Despite the proliferation of bibliometric studies in other disciplines, there is a lack of large-scale, field-specific analyses in orthopaedics that examine the combined influence of authorship patterns, title features, and OA status on citation performance using a comprehensive dataset spanning multiple years. This study evaluated whether the number of authors, title characteristics, study design and OA status influence the citation rate of orthopaedic research articles.

## Methods

### Data source

We retrieved the list of orthopaedic publications from Scopus through a keyword-based search, providing digital object identifier (DOI), publication year, author lists, article titles and total citations. The database was queried programmatically via DOI-based requests, and the citation count returned was stored as a numeric variable. OA status was returned as a Boolean value and stored without transformation until recoding for statistical analysis. Scopus was accessed between 1 January 2010 and 31 December 2020, ensuring consistency in citation counts and OA status across the dataset.

### Search strategy

The bibliographic dataset was produced from a structured search of Scopus. The search query consisted of the keyword ‘orthopaedic’ applied to all searchable fields, without filters for study type, language or publication status. The search was executed iteratively to manage export limits, with results retrieved in annual batches covering the publication years 2010 to 2020 inclusive. The search was limited to 10,000 records per year. The DOI and associated metadata were exported directly from Scopus for each record. Metadata fields included publication year, complete author list, citation count as indexed in Scopus, and OA status. The number of authors for each article was calculated from the complete author list. OA status was stored as a binary variable, with ‘true’ for OA articles and ‘false’ for non-OA articles. A separate metadata file containing the DOI and corresponding article title was created in parallel and sourced from the same Scopus export. The title file contained no duplicate DOIs; titles were stored in their original format as indexed in Scopus. The bibliographic and title tables were exported in comma-separated values (CSV) format for subsequent integration and analysis. The two datasets were kept separate until the integration stage of the analysis, described in the ‘Statistical analyses’ section, to reduce file size during initial processing and to minimise error propagation during the enrichment of bibliographic variables.

### Statistical analyses

Two CSV files were imported into Python (Python Software Foundation, version 3.11) using the Pandas library (pandas 2.x, pandas.pydata.org). Records with missing information or duplicates were removed. All variable types were standardised: Publication year was cast to an integer; number of authors and citation counts were converted to numeric; and OA status was converted to a Boolean where possible. Derived variables were calculated for subsequent analysis. ‘Years since publication’ was defined as the difference between the reference year (2025) and the publication year, plus one to account for the publication year itself. The primary dependent variable, ‘citations_per_year’, was calculated as total citation count divided by years since publication, providing a time-normalised measure of citation rate. Additionally, a within-year citation percentile (‘percentile_per_year’) was computed by grouping articles by publication year, ranking citation counts in ascending order and expressing each rank as a percentage of the total records for that year. Two multiple linear regression models were then specified using the statsmodels library with ordinary least squares (OLS) estimation. The first model included ‘num_authors’ (continuous) and ‘is_oa’ (binary indicator, with 1 for OA and 0 for non-OA) as independent variables, with ‘citations_per_year’ as the dependent variable. The second model examined the influence of title characteristics on ‘citations_per_year’. For this model, title-derived predictors were computed programmatically: ‘title_length_chars’ (string length in characters, including spaces and punctuation), ‘title_length_words’ (number of whitespace-separated tokens) and three binary indicators representing the presence of a colon (‘:’), a question mark (‘?’) and a dash (‘-’). These binary variables were assigned a value of 1 if the symbol was present and zero otherwise. In addition to these title-derived predictors, the capitalisation style of article titles was also evaluated. Each title was programmatically classified into one of four categories: ‘all_caps’ when all alphabetic characters were uppercase, ‘title_case’ when the majority of words began with an uppercase initial, ‘all_lower’ when the majority of words were lowercase and ‘mixed’ for titles not meeting the previous criteria. Articles that could not be reliably categorised were assigned to an ‘unknown’ group and excluded from inferential testing. Differences in time-normalised citation rates across capitalisation categories were tested using one-way analysis of variance (ANOVA) and confirmed with the non-parametric Kruskal–Wallis test to account for skewed citation distributions. Before model fitting, observations with missing or invalid values (NaN or infinite) in either the dependent variable or the predictors included in a given model were excluded listwise. No transformations, scaling or interaction terms were applied to the predictors, and all variables were entered simultaneously into each model. All tests were two-sided, and the statistical significance threshold was set at *p* < 0.05. Model fit was evaluated using the coefficient of determination (*R*^2^) and adjusted *R*^2^. Using an exploratory modelling approach, subgroup analyses were conducted to identify the combination of author number and title characteristics most strongly associated with citation rates. Starting from the integrated dataset, additional variables were derived from article titles: total length in characters; length in words; binary indicators for the presence of a colon, a question mark and a dash; and the categorical classification of capitalisation style. A multiple linear regression model was then refitted using ordinary least squares estimation, incorporating quadratic terms for the continuous variables (number of authors, title length in characters and title length in words) to account for potential non-linear associations with citation rate. This model calculated predicted citation rates across a predefined grid of plausible values for each predictor: number of authors from 1 to 20, title length from 30 to 200 characters, title length from 5 to 25 words and all combinations of punctuation and capitalisation indicators. The grid was evaluated using the fitted regression equation, and the combination yielding the highest predicted citation rate was recorded as the optimal configuration within the observed data range. The type of study design of each article was extracted and classified on the basis of predefined terms to categorise articles into the following groups: randomised controlled trial, clinical trial, observational study, systematic review, network meta-analysis, meta-analysis, narrative review, case report, case series, technical note, editorial, and commentary. Classification was performed hierarchically to avoid overlap, for example, assigning articles containing ‘network meta-analysis’ separately from those labelled ‘meta-analysis’ or ‘systematic review’. Articles not matching the specified criteria were coded as ‘unclassified’. Time-normalised citation rates were then compared across study design categories. Overall differences were assessed using the Kruskal–Wallis test, given the skewed distribution of citation counts, and pairwise comparisons were performed using Mann–Whitney *U* tests with Bonferroni correction to adjust for multiple testing. Statistical significance was set at *p* < 0.05.

## Results

### Search results

The bibliographic search identified 97,806 orthopaedic articles published between 2010 and 2020, for which DOIs, publication year, number of authors, citation counts, titles and OA status were all available directly from Scopus. The export was performed in annual batches to accommodate download limits, then combined into a single dataset. Duplicate DOIs, which arose from multiple indexed entries associated with the same identifier, were identified and resolved by retaining the first occurrence, resulting in the removal of 1921 records. The final integrated dataset contained 97,806 unique articles, each with complete bibliographic, citation and OA information. This dataset was used directly for descriptive statistics and was the basis for all subsequent regression analyses.

### Generalities

The final dataset comprised 97,806 unique orthopaedic articles published between 2010 and 2020. The number of authors per article ranged from single-authored articles to significant multi-authored works, with a mean of 5.76 authors and a median of 5. OA status was evenly distributed, with 50.1% of the articles classified as OA. Citation counts showed a right-skewed distribution, with a mean of 31.97 citations and a median of 16. When adjusted for the years since publication, the mean citation rate was 2.96 citations per year, and the median was 1.6.

### Association between the number of authors, OA and citation rate

A multiple linear regression model was fitted with the time-normalised citation rate (citations per year since publication) as the dependent variable and the number of authors and OA status as predictors (Table 1). OA status was coded as 1 for OA and 0 for non-OA articles. The analysis included 94,720 articles with complete data for all variables. The model intercept was 2.30 (95% confidence interval [CI] 2.22 to 2.38, *p* < 0.001). The coefficient for the number of authors was 0.108 (95% CI 0.103 to 0.114, *p* < 0.001), indicating that each additional author was associated with an increase of 0.108 citations per year. The coefficient for OA status was 0.175 (95% CI 0.071 to 0.279, *p* = 0.001), corresponding to an increase of 0.175 citations per year for OA articles compared with non-OA articles. The model explained 1.79% of the variance in citation rate (*R*^2^ = 0.0179, adjusted *R*^2^ = 0.0179).

**Table 1:** Results of the analysis on the association between the number of authors, OA and citation rate

Variable	Coefficient	95% CI	*p*-Value	Interpretation
Intercept	2.30	2.22 to 2.38	< 0.001	Expected citations/year for a reference article
Number of authors	0.108	0.103 to 0.114	< 0.001	+0.108 citations/year for each additional author
Open access (OA versus non)	0.175	0.071 to 0.279	0.001	+0.175 citations/year for OA articles versus non-OA

### Association between title characteristics and citation rate

A multiple linear regression model was fitted with the time-normalised citation rate (citations per year since publication) as the dependent variable and title characteristics as predictors (Table 2). Independent variables included title length in characters, title length in words and binary indicators for the presence of a colon (‘:’), a question mark (‘?’) and a dash (‘-’). The model intercept was 2.97 (95% CI 2.87 to 3.06, *p* < 0.001). Title length in characters was positively associated with the citation rate (coefficient 0.023, 95% CI 0.019 to 0.027, *p* < 0.001), whereas title length in words showed a negative association (coefficient −0.183, 95% CI −0.213 to −0.154, *p* < 0.001). The presence of a colon was associated with an increase of 0.314 citations per year (95% CI 0.200 to 0.428, *p* < 0.001), the presence of a question mark was associated with a decrease of 0.476 citations per year (95% CI −0.724 to −0.227, *p* < 0.001) and the presence of a dash was associated with an increase of 0.187 citations per year (95% CI 0.073 to 0.301, *p* = 0.001). The model explained 0.23% of the variance in citation rate (*R*^2^ = 0.00235, adjusted *R*^2^ = 0.00230). When examining the influence of title capitalisation style, 62,433 articles were classified into one of four categories: 15,965 as title case, 44,469 as all lower, 499 as all capitals and 35,037 as mixed, while 2052 records could not be reliably categorised and were excluded from inferential testing. Descriptive analysis showed that articles in all capitals had markedly fewer citations per year (mean 0.71, median 0.44) compared with title case (mean 3.04, median 1.67), all lower (mean 3.02, median 1.69) and mixed titles (mean 2.99, median 1.54). One-way ANOVA indicated a statistically significant difference between groups (*F* = 13.27, *p* < 0.000000012), which the Kruskal–Wallis test confirmed (*H* = 604.05, *p* ≈ 1.3 × 10^−130^). Post hoc comparisons revealed that the lower citation rate of all capitalized titles accounted for the overall significance. In contrast, no substantial difference was observed between title case and all lower categories. These findings suggest that using all capital letters in titles is associated with significantly reduced citation performance. In contrast, the distinction between conventional title case and lower case styles does not appear to influence citation rates.

**Table 2:** Results of the analysis on the association between title characteristics and citation rate

Variable	Coefficient	95% CI	*p*-Value	Interpretation
Intercept	2.97	2.87 to 3.06	< 0.001	Expected citations/year for a reference title
Title length (per character)	0.023	0.019 to 0.027	< 0.001	+0.023 citations/year for each additional character in the title
Title length (per word)	−0.183	−0.213 to −0.154	< 0.001	−0.183 citations/year for each additional word in the title
Colon ‘:’ present	0.314	0.200 to 0.428	< 0.001	+0.314 citations/year if colon included in the title
Dash ‘-’ present	0.187	0.073 to 0.301	0.001	+0.187 citations/year if dash included in the title
Question mark ‘?’ present	−0.476	−0.724 to −0.227	< 0.001	−0.476 citations/year if question mark included in the title

### Association between the number of authors and citation rate

Exploratory non-linear modelling (Table 3) showed that the predicted citation rate increased steadily with the number of authors, reaching its highest value of 8.38 citations per year at the maximum tested team size of 20 authors. As the curve did not approach a plateau within this range, no cut-off for an optimal author count could be determined. For title characteristics, the model indicated an optimal length of approximately 195 characters (predicted 3.63 citations/year) and five words (predicted 4.87 citations/year) when other factors were held constant. When all predictors were combined, the configuration associated with the highest predicted citation rate was a 20-author article with a title of around 200 characters and five words, containing a colon and a dash but no question mark, corresponding to an estimated 11.25 citations per year.

**Table 3:** Results of the analysis on the association between the number of authors and citation rate

Predictor	Optimal value	Predicted citations/year	Comments
Number of authors	20 (maximum tested)	8.38	Citation rate steadily increases with more authors; no plateau observed
Title length (characters)	Approximately 195	3.63	Optimal number of title characters for the highest citation prediction
Title length (words)	Approximately 5	4.87	The optimal title word count is low for the best citation rate
Combined configuration	20 authors; ~200 characters; 5 words; colon and dash present; no question mark	11.25	This combination predicts the highest citation rate

### Association between study design and citation rate

When comparing study designs, apparent differences in citation performance emerged (Table 4). Network meta-analyses (*n* = 60) achieved the highest citation rate, with a mean of 6.64 citations per year (median 3.07), followed by systematic reviews (*n* = 1378; mean 5.66, median 3.72), meta-analyses (*n* = 1183; mean 5.08, median 3.18) and narrative reviews (*n* = 1744; mean 4.81, median 2.43). Randomised controlled trials (*n* = 701) and clinical trials (*n* = 351) performed at an intermediate level, with means of 3.90 and 3.86 citations per year and medians of 2.36 and 2.14, respectively. Observational studies (*n* = 197) showed a lower impact (mean 2.40, median 1.60), followed by case series (*n* = 438; mean 1.79, median 1.36), technical notes (*n* = 94; mean 1.33, median 0.83) and case reports (*n* = 2987; mean 0.77, median 0.50). Editorials (*n* = 173) and commentaries (*n* = 128) had the lowest citation performance, with mean values of 0.51 (median 0.09) and 0.25 (median 0.08) citations per year, respectively. The Kruskal–Wallis test confirmed significant overall differences among study types (*H* = 4549.56, *p* < 0.0001). Pairwise post hoc analyses with Bonferroni correction revealed that high-level evidence studies, such as network meta-analyses, systematic reviews and meta-analyses, were consistently cited more frequently than low-level designs, particularly case reports, commentaries and editorials (all *p* < 0.001). In contrast, no significant difference was detected between randomised controlled and clinical trials (*p* > 0.05), indicating comparable citation performance between these categories.

**Table 4:** Results of the analysis on the association between study design and citation rate

Study design	Size (*n*)	Mean citations/year	Median citations/year	Citation rank	Interpretation
Network meta-analyses	60	6.64	3.07	1 (highest)	Highest impact; integrates multiple treatments, widely cited for evidence synthesis
Systematic reviews	1378	5.66	3.72	2	Highly cited secondary research summarising evidence
Meta-analyses	1183	5.08	3.18	3	Synthesises pooled data, valued for comprehensive insights
Narrative reviews	1744	4.81	2.43	4	Less rigorous than systematic reviews, but practical summaries
RCT	701	3.90	2.36	5 (intermediate)	Primary high-quality evidence, robust methodology, but a narrower scope than reviews
Clinical trials	351	3.86	2.14	6 (intermediate)	Necessary primary research, similar citation impact to RCTs
Observational studies	197	2.40	1.60	7	Valuable for real-world data, but less citation impact owing to lower evidence level
Case series	438	1.79	1.36	8	Lower evidence level, descriptive clinical data
Technical notes	94	1.33	0.83	9	Brief reports of technical innovations, modest citation impact
Case reports	2987	0.77	0.50	10 (low)	Hypothesis-generating, but with the lowest citation rates
Editorials	173	0.51	0.09	11 (lowest)	Opinion pieces, minimal original data, low citation impact
Commentaries	128	0.25	0.08	12 (lowest)	Similar to editorials, the least cited article types

## Discussion

This investigation explores how certain bibliometric variables and features of article titles relate to the rate at which orthopaedic publications accumulate citations over time. The present study’s findings suggest that articles with more authors and those published in OA tend to receive more citations each year. However, these factors alone explain only a small part of the variation observed. This pattern aligns with the idea that wider collaborative networks may increase the visibility of a study and that OA availability can facilitate access and readership [13, 14, 18]. At the same time, the limited proportion of variance explained points to the fact that citation performance is influenced by a complex interplay of factors, mostly beyond the scope of this analysis. Analysing the features of article titles, some consistent patterns emerged. Titles containing more characters were associated with slightly higher citation rates, whereas titles containing more words tended to be cited less frequently. One possible explanation is that concise but information-rich titles capture attention more effectively than those with many shorter words, which can appear less focused. Certain punctuation marks also appeared to matter: Titles including a colon or a dash were associated with modestly higher citation rates, possibly reflecting how these devices are often used to introduce specificity or highlight a key finding. Conversely, a question mark was linked with lower citation rates, a trend noted in other fields, and might be related to perceptions of uncertainty or a less definitive contribution. While these associations were statistically significant, the very low explanatory power of the model suggests that title construction is only one small element in the complex set of factors influencing citation performance. The optimisation analysis offers a data-driven illustration of how certain authorship and title features may influence citation performance in orthopaedic research. The steady rise in predicted citation rate with increasing author numbers, without evidence of a plateau within the tested range, suggests that larger collaborative teams tend to achieve greater visibility, possibly through broader professional networks and wider dissemination of findings. Title-related factors showed a preference for concise, information-rich phrasing, with the model identifying a configuration of approximately 200 characters and only five words, supplemented by punctuation such as a colon and a dash, but avoiding question marks, as the combination most strongly associated with higher citation rates. While these findings are statistically derived and cannot be interpreted as causal, they align with the broader bibliometric literature. This indicates that collaborative scale and careful title construction can be measurable in maximising scholarly reach. The analysis of capitalisation styles in article titles further highlighted the potential influence of formatting choices on citation performance. Titles written entirely in uppercase were associated with significantly lower citation rates than other styles. This suggests that this format may reduce readability or be perceived as less professional within the academic community. In contrast, no meaningful difference was detected between conventional title case and lowercase styles, which performed similarly with respect to citation impact. These findings align with broader evidence indicating that clarity and conventional formatting enhance discoverability and acceptance, whereas non-standard presentation may limit scholarly uptake [12, 16]. Title capitalisation is often dictated by a journal’s ‘Instructions for Authors’ and editorial style guides rather than by authors’ deliberate choices. Consequently, the capitalisation format may act as a proxy for journal-level policies or historical formatting conventions, rather than representing an intrinsic characteristic of the article or its authors. While the effect size remains modest compared with other determinants such as collaboration and open access, optimising title presentation is a simple modifiable factor that authors can consider to improve the visibility of their work [24, 25]. The findings clearly show that not all study types attract the same level of attention in the scientific community. As expected, network meta-analyses, systematic reviews and meta-analyses were cited far more frequently than other categories, which reflects their role as reference points for clinicians and researchers. Randomised controlled and clinical trials occupied an intermediate position, confirming their central role as primary sources of evidence. However, individual trials often have a narrower readership compared with secondary research. At the other end of the spectrum, case reports, case series, technical notes and editorials had much lower citation counts. This is not surprising, since these formats are usually hypothesis-generating or educational rather than definitive evidence. Yet, their limited citation performance should not diminish their scientific or clinical value. Many important medical discoveries have begun with case observations, and technical notes often yield practical innovations that directly shape surgical practice [26]. Thus, while our analysis quantifies the citation patterns associated with study design, it is essential to appreciate that scientific progress relies on the interplay of all these contributions, regardless of their immediate impact.

Although the findings offer measurable associations between bibliometric features and citation rates, they are inherently scientometric and must be interpreted within the broader context of sound research practice. Certain variables, such as the article title, can be consciously refined to optimise clarity, focus and potential discoverability without compromising scientific integrity. By contrast, authorship should adhere to recognised guidelines, such as the International Committee of Medical Journal Editors (ICMJE) [27, 28]. It should reflect substantial intellectual or practical contributions rather than aiming to maximise citation potential. The observation that larger teams are associated with higher citation rates does not justify inflating author lists beyond legitimate contributions, as this would undermine both ethical standards and the work’s credibility.

Although it shows a modest yet significant association with increased citation rates, the role of OA warrants careful consideration. OA publishing can broaden readership and accelerate knowledge dissemination, yet its sustainability is debated. The prevailing article processing charge (APC) model places substantial financial responsibility on authors or their institutions, which may exacerbate inequalities in global research participation. Furthermore, the notion that scientific knowledge must be paid for at high cost to be accessed or published is increasingly questioned, particularly in light of initiatives that promote publicly funded, freely available research. As such, while OA can contribute to scholarly reach, its implementation should be balanced against ethical considerations of equity, inclusivity and the core principle that research should serve the public interest rather than market imperatives.

This study provides important insights into the factors that influence the visibility and impact of orthopaedic research publications. The analysis of nearly 100,000 articles highlights how collaboration, open access and carefully structured titles can enhance citation performance. These findings have direct practical implications for clinicians and researchers aiming to optimise the dissemination of their work. For instance, adopting ethical collaborative practices, considering open access routes where feasible and crafting concise, information-rich titles can improve the reach and scholarly impact of their research. Moreover, the clear citation advantage of high-level evidence designs such as systematic reviews and meta-analyses reinforces the importance of rigorous study design in shaping scientific influence. Thus, this study helps guide researchers in making informed choices that may increase the clinical and academic visibility of their contributions without compromising scientific integrity.

Despite its large dataset and robust statistical analyses, the study has inherent limitations that must be acknowledged. First, citation counts, although widely used, are an imperfect proxy for scientific quality, as they can be influenced by factors unrelated to methodological rigour, such as trends, self-citation or field size. Second, the reliance on a single database (Scopus) may limit generalisability, as different indexing services can yield variable results. The explanatory power of the models was relatively low, indicating that many determinants of citation performance lie beyond the bibliometric features examined. Additionally, while associations were identified, causal inferences cannot be drawn. For example, a longer title may correlate with higher citation rates, but this does not mean it directly drives them. Finally, the study is limited to articles published between 2010 and 2020, and citation dynamics may evolve with changes in publishing models, digital platforms and research practices.

While scientometric analyses provide valuable insights into the factors associated with citation performance, it is essential to recognise that the ultimate purpose of scientific endeavour extends beyond visibility metrics. Academic impact measured through citations can inform strategies to optimise dissemination, yet it should never become the primary aim of research. The true responsibility of scholars is to pursue rigorous, ethical and innovative science that advances knowledge, improves patient care and contributes positively to society [28]. Citations may reflect reach but do not fully capture quality, originality or integrity. Therefore, while this study highlights modifiable elements of publishing practice, it must be emphasised that genuine scientific progress rests on methodological soundness, transparent reporting and adherence to ethical principles, which remain more important than any quantitative indicator of success.

## Conclusions

In orthopaedic research, larger collaborative teams, OA availability and concise, character-rich titles with selected punctuation were each associated with higher citation rates. While title formatting can be adjusted to enhance discoverability, authorship should remain consistent with established ethical guidelines, and the implications of OA publishing models warrant careful consideration. In addition, unconventional formatting choices, such as using all capital letters in titles, are associated with reduced citation performance, whereas conventional title case and lower case styles yield comparable outcomes. Network meta-analyses, systematic reviews and meta-analyses achieved the highest citation rates, while randomised controlled trials and clinical trials occupied an intermediate position. In contrast, case reports, case series, technical notes, editorials and commentaries consistently showed lower citation performance. While citation performance offers valuable insights, genuine scientific value lies in rigorous, ethical and meaningful research that advances knowledge beyond mere metrics.

## Data Availability

The datasets generated during and/or analysed during the current study are available throughout the manuscript.

## References

[1] Aman V, Gläser J (2025) Investigating knowledge flows in scientific communities: the potential of bibliometric methods. Minerva 63(1):155–182

[2] Abramo G, D'Angelo CA (2024) Analyzing the inter-domain versus intra-domain knowledge flows. arXiv preprint arXiv:240401818

[3] Vaishya R, Gupta BM, Mamdapur GMN, Vaish A, Migliorini F (2023) Scientometric analysis of highly cited papers on avascular necrosis of the femoral head from 1991 to 2022. J Orthop Traumatol 24(1):27. 10.1186/s10195-023-00709-3 37322138 10.1186/s10195-023-00709-3 PMC10272038

[4] Li Y, Xu G, Long X, Ho YS (2019) A bibliometric analysis of classic publications in web of science category of orthopedics. J Orthop Surg Res 14(1):227. 10.1186/s13018-019-1247-1 31324248 10.1186/s13018-019-1247-1 PMC6642495

[5] Vaishya R, Shekhawat S, Vaish A, Migliorini F (2025) Evolution of journal rankings in orthopedics and sports medicine (2000–2024): a SCImago-based bibliometric analysis. Orthopadie (Heidelb). 10.1007/s00132-025-04683-y 40745495 10.1007/s00132-025-04683-y PMC12457506

[6] Vaishya R, Vaish A, Schafer L, Migliorini F (2024) Publications and ranking in orthopaedics and sports medicine of European countries during the last three decades: a bibliometric analysis. J Orthop 58:96–101. 10.1016/j.jor.2024.07.002 39100540 10.1016/j.jor.2024.07.002 PMC11292423

[7] Waltman L (2016) A review of the literature on citation impact indicators. J Informetr 10(2):365–391

[8] Aksnes DW, Langfeldt L, Wouters P (2019) Citations, citation indicators, and research quality: an overview of basic concepts and theories. SAGE Open 9(1):2158244019829575

[9] Pierannunzii L, d’Imporzano M (2014) The journal of orthopaedics and traumatology in the 15th year from foundation: actual achievements and future directions. J Orthop Traumatol 15(4):235–238. 10.1007/s10195-014-0326-7 25403927 10.1007/s10195-014-0326-7 PMC4244542

[10] Colavizza G, Cadwallader L, LaFlamme M, Dozot G, Lecorney S, Rappo D, Hrynaszkiewicz I (2024) An analysis of the effects of sharing research data, code, and preprints on citations. PLoS ONE 19(10):e0311493 39475849 10.1371/journal.pone.0311493 PMC11524460

[11] Falagas ME, Zarkali A, Karageorgopoulos DE, Bardakas V, Mavros MN (2013) The impact of article length on the number of future citations: a bibliometric analysis of general medicine journals. PLoS ONE 8(2):e49476 23405060 10.1371/journal.pone.0049476 PMC3566179

[12] Jamali HR, Nikzad M (2011) Article title type and its relation with the number of downloads and citations. Scientometrics 88(2):653–661

[13] Gazni A, Sugimoto CR, Didegah F (2012) Mapping world scientific collaboration: authors, institutions, and countries. J Am Soc Inf Sci Technol 63(2):323–335

[14] Wuchty S, Jones BF, Uzzi B (2007) The increasing dominance of teams in production of knowledge. Science 316(5827):1036–1039 17431139 10.1126/science.1136099

[15] Bornmann L, Daniel HD (2008) What do citation counts measure? A review of studies on citing behavior. J Doc 64(1):45–80

[16] Paiva CE, Lima JPdSN, Paiva BSR (2012) Articles with short titles describing the results are cited more often. Clinics 67:509–513 22666797 10.6061/clinics/2012(05)17 PMC3351256

[17] Li Y, Xu G, Long X, Ho Y-S (2019) A bibliometric analysis of classic publications in web of science category of orthopedics. J Orthop Surg Res 14(1):227 31324248 10.1186/s13018-019-1247-1 PMC6642495

[18] Piwowar H, Priem J, Larivière V, Alperin JP, Matthias L, Norlander B, Farley A, West J, Haustein S (2018) The state of OA: a large-scale analysis of the prevalence and impact of open access articles. PeerJ 6:e4375 29456894 10.7717/peerj.4375 PMC5815332

[19] Eger T, Mertens A, Scheufen M (2021) Publication cultures and the citation impact of open access. Manag Decis Econ 42(8):1980–1998

[20] Eysenbach G (2006) Citation advantage of open access articles. PLoS Biol 4(5):e157 16683865 10.1371/journal.pbio.0040157 PMC1459247

[21] D’Ambrosi R, Migliorini F, Di Maria F, Anghilieri FM, Di Feo F, Ursino N, Mangiavini L, Kambhampati SBS (2024) Italian research on anterior cruciate ligament: a bibliometric analysis. Eur J Orthop Surg Traumatol 34(5):2235–2243. 10.1007/s00590-024-03937-3 38602582 10.1007/s00590-024-03937-3

[22] Tennant JP, Waldner F, Jacques DC, Masuzzo P, Collister LB, Hartgerink CH (2016) The academic, economic and societal impacts of open access: an evidence-based review. F1000Res 5:632. 10.12688/f1000research.8460.3 27158456 10.12688/f1000research.8460.1 PMC4837983

[23] Solomon DJ, Björk BC (2012) A study of open access journals using article processing charges. J Am Soc Inf Sci Technol 63(8):1485–1495

[24] Zahedi Z, Haustein S (2018) On the relationships between bibliographic characteristics of scientific documents and citation and Mendeley readership counts: a large-scale analysis of Web of Science publications. J Informetr 12(1):191–202

[25] Sienkiewicz J, Altmann EG (2016) Impact of lexical and sentiment factors on the popularity of scientific papers. R Soc Open Sci 3(6):160140 27429773 10.1098/rsos.160140 PMC4929908

[26] Vandenbroucke JP (2001) In defense of case reports and case series. Ann Intern Med. 10.7326/0003-4819-134-4-200102200-00017 11182844 10.7326/0003-4819-134-4-200102200-00017

[27] Defining the role of authors and contributors. (2024) International Committee of Medical Journal Editors. https://www.icmje.org/recommendations/browse/roles-and-responsibilities/defining-the-role-of-authors-and-contributors.html. Accessed 06.09.2025

[28] Migliorini F, Randelli F, Di Martino A, Rivera F (2025) Writing for JOOT: raising standards in clinical research and evidence synthesis. J Orthop Traumatol 26(1):52. 10.1186/s10195-025-00868-5 40760235 10.1186/s10195-025-00868-5 PMC12321706

